# Supplementary material for: Structure-based discovery of small molecules that disaggregate Alzheimer’s disease tissue derived tau fibrils in vitro
Source: Nat Commun. 2022 Sep 16;13:5451. doi: 10.1038/s41467-022-32951-4 (PMC9481533; doi:10.1038/s41467-022-32951-4)
Supplement: Supplementary file 1 — Supplementary Information [file 41467_2022_32951_MOESM1_ESM.docx]

**Structure-based discovery of small molecules that disaggregate tau fibrils from Alzheimer’s disease**

Paul M. Seidler^1,2,3,4,5,6^ Kevin A. Murray^1,2,3,4,5^, David R. Boyer^1,2,3,4,5^, Peng Ge^1,2,3,4,5^, Michael R. Sawaya^1,2,3,4,5^, Carolyn J. Hu^1,2,3,4,5^, Xinyi Cheng^1,2,3,4,5^, Romany Abskharon^1,2,3,4,5^, Hope Pan^1,2,3,4,5^, Michael A. DeTure^7^, Christopher K. Williams^8^, Dennis W. Dickson^7^, Harry V. Vinters^8,9^, David S. Eisenberg^1,2,3,4,5,6,*^

^1^Department of Chemistry and Biochemistry, UCLA. ^2^Department of Biological Chemistry, UCLA. ^3^UCLA-DOE Institute. ^4^Molecular Biology Institute, UCLA. ^5^Howard Hughes Medical Institute. ^6^University of Southern California, Department of Pharmacology and Pharmaceutical Sciences, Los Angeles, CA. ^7^Department of Neuroscience, Mayo Clinic, Jacksonville, Florida. ^8^Department of Pathology and Laboratory Medicine, David Geffen School of Medicine, UCLA, Los Angeles CA 90095. ^9^Department of Neurology, David Geffen School of Medicine, UCLA, Los Angeles, CA 90095. ✉email: [david@mbi.ucla.edu](mailto:david@mbi.ucla.edu)

These authors contributed equally to this work: Paul M. Seidler, Kevin A. Murray, David R. Boyer.

**Supplementary Information**

**Supplementary Fig. 1: Time-resolved disaggregation of brain-derived tau PHFs by EGCG.** **a-b.** Brain derived tau PHFs were incubated with 40 µM EGCG at various timepoints, then analyzed by dot blot and probed with two tau antibodies. **a**. The monoclonal antibody GT38 specifically recognizes pathological tau aggregates. A decrease in GT38 staining is observed with increasing EGCG incubation time, indicating a reduction in AD tau fibrils. **b.** The AT8 antibody recognizes total hyperphosphorylated tau (both aggregate and monomeric), and no change is seen in total tau signal with EGCG treatment. Error bars indicate ± SD, experiments performed in triplicate. Statistical significance was analyzed by one way ANOVA (**p<0.05) of n=3 technical replicates.


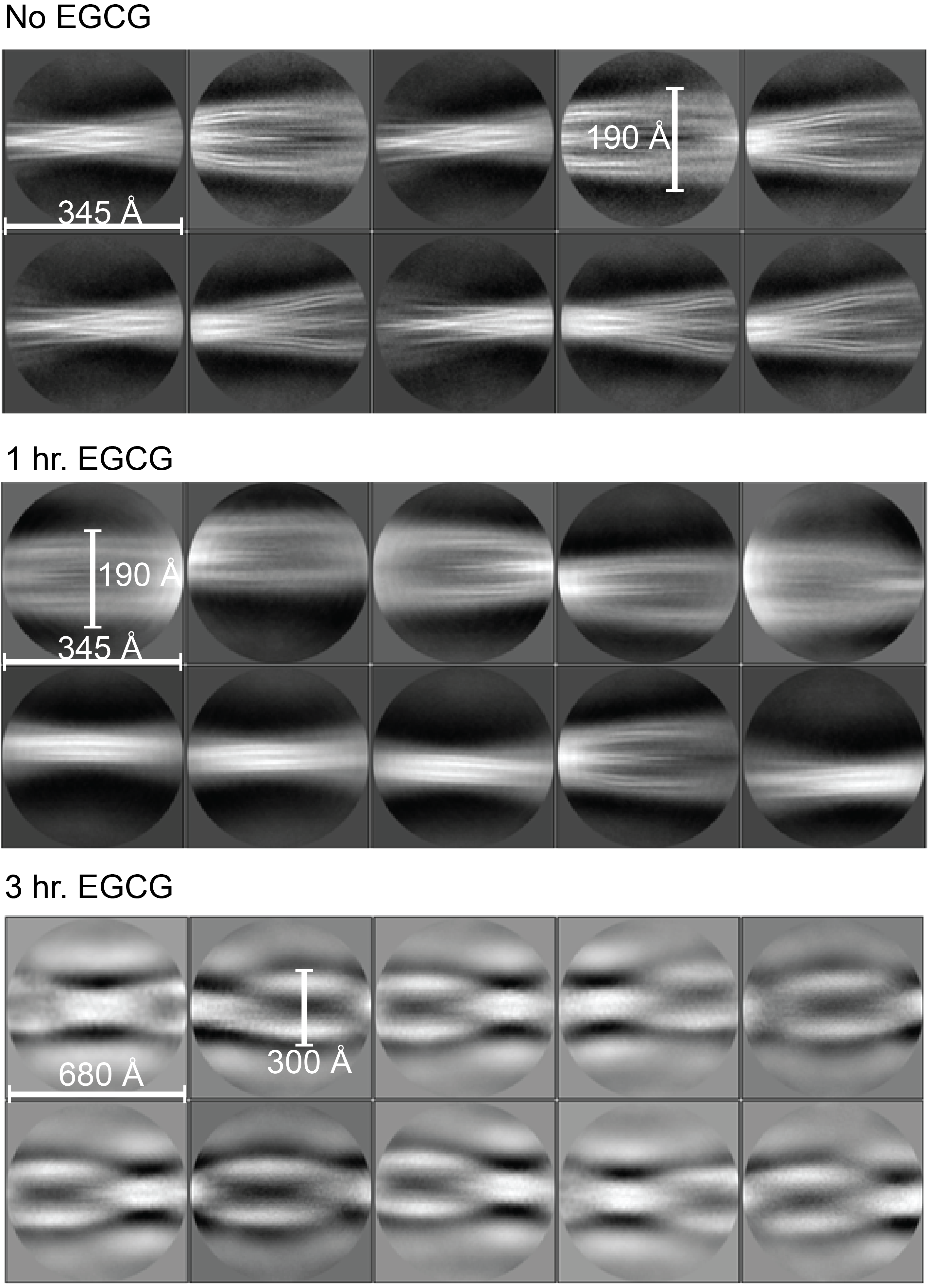


**Supplementary Fig. 2: 2D class averages of no EGCG, 1-hour EGCG, and 3-hour EGCG incubation.** At 3 hours, EGCG incubation results in less strongly defined features of the 2D class averages, perhaps as a result of tau fibril destabilization.

**
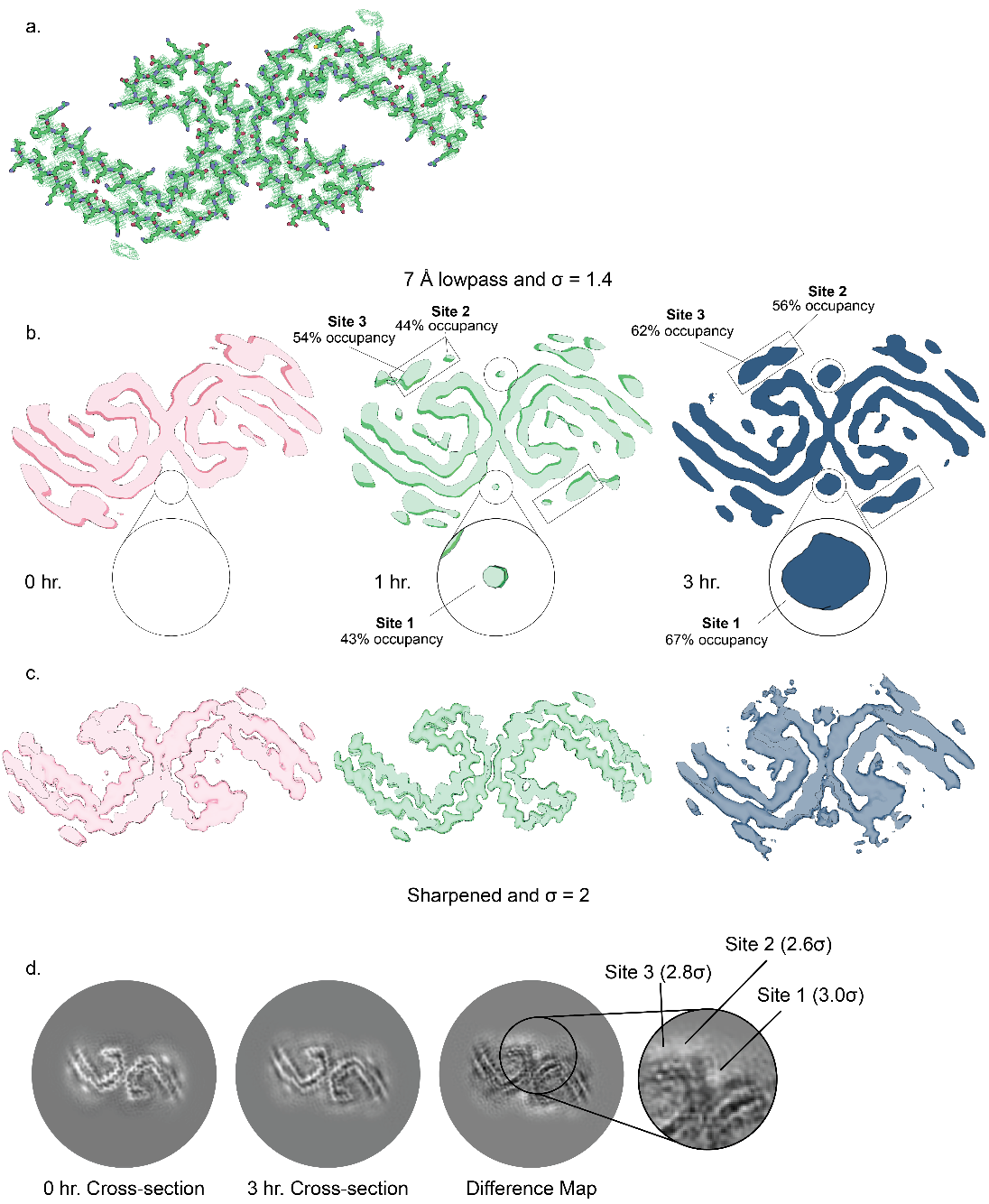
**

**Supplementary Fig. 3: Tau PHF structure incubated with EGCG at multiple timepoints. a.** As part of our kinetic study of EGCG binding to tau, we determined the 3.3 Å structure of the PHF in complex with EGCG following a 1-hour incubation period. The final, sharpened density map of the structure does not reveal the EGCG density observed in the 3-hour structure. **b**. Time-resolved cryoEM density maps low-pass filtered to 7 Å. Site 1 is circled while Sites 2-3 are boxed. After lowpass filtering all three structures to 7 Å, at 0-hour, no density is observed (pink); at 1-hour (green), density at Sites 1-3 is observed with Sites 1-3 having 43%, 44%, and 54% the occupancy of the fibril backbone, respectively; at 3 hr., the maximum density at Sites 1-3 is observed, with Sites 1-3 having 67%, 56%, and 62% of the occupancy of the fibril backbone (blue). Notably, the increase in density at Sites 1-3 corresponds with the disaggregation of fibrils as observed by negative stain EM (Figure 1b) and dot blot (Supplementary Fig. 1) **c**. When fully sharpened, only the 3-hour structure (blue) shows new density for EGCG at Sites 1-3. **d.** X-Y cross-sections of central rung of 0 and 3 hr. fibrils and corresponding difference map. The difference map shows that EGCG sites 1-3 are 3, 2.6, and 2.8 σ above the noise level, respectively. Some protein backbone features are present in the difference map due to differing resolutions of the 0 hr. and 3 hr. map, as well as subtle backbone changes between the two maps. Interestingly, the densities found flanking K311/K317/K321 that are present in all AD tau fibril structures, including our 0 hr. and 3 hr. structures (densities seen at bottom left and top right of the cross-sections) are eliminated in the difference map. This demonstrates that we captured known additional densities in our structures and that they are equally present in both our 0 and 3 hr. structures, serving as an internal control.**
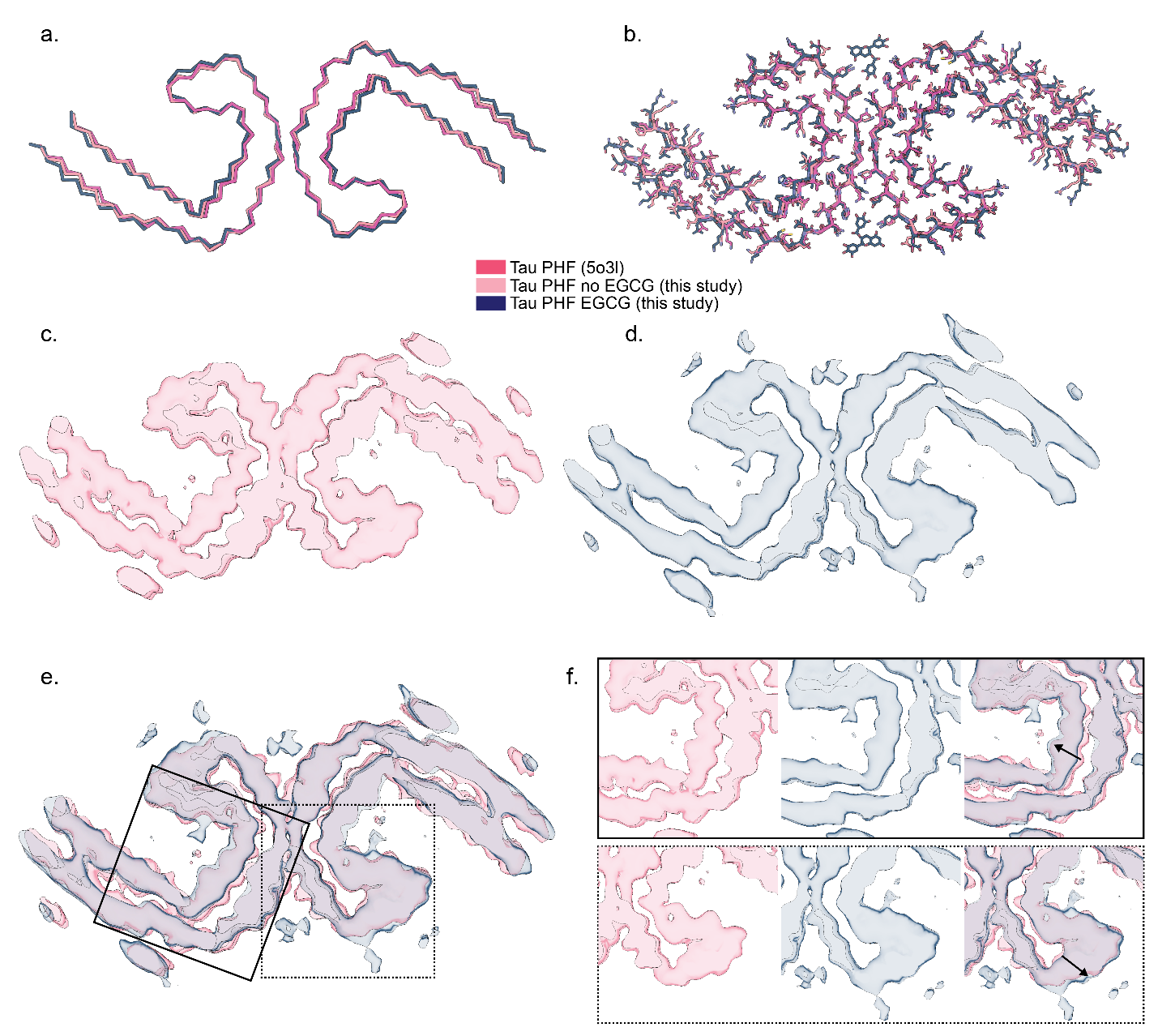
Supplementary Fig. 4: Comparison of tau PHF structures and density maps with and without EGCG bound. a.** Backbone overlay of tau PHF without EGCG (pink), the previously published tau PHF, and tau PHF in complex with EGCG (blue). The no EGCG and 3-hour EGCG structures determined here are nearly identical to the previously published structure (pdb 5o3l). **b.** Overlay of the three PHF structures with sidechains visible highlights the similarity between each structure. **c-f**. Density maps of the PHF without EGCG (**c**) and with EGCG (**d**). **e-f**. Two regions of the fibrils show slight shifts in the density maps as a result of EGCG binding (highlighted in black boxes). **f**. These shifts are observed near the C-shaped cavity of the fibril core (top) and the beta-helix region (bottom), in which the density of EGCG-bound structure (blue) slightly shifts radially outward, which may represent an allosteric affect from EGCG binding or the beginnings of fibril destabilization. Black arrows indicate regions with slight shifts in density between bound and unbound structures.


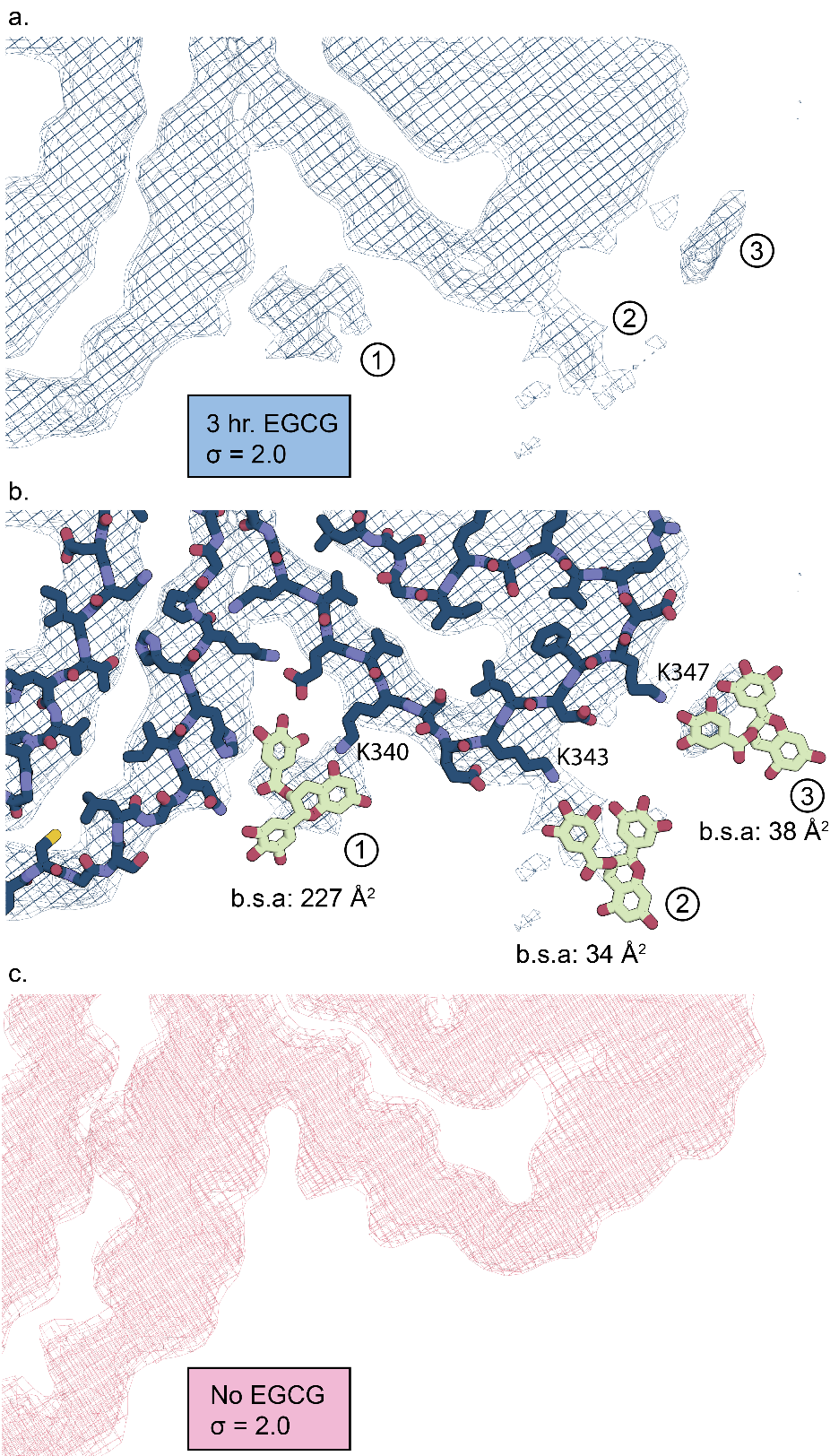


**Supplementary Fig. 5: Three possible binding sites of EGCG on the tau PHF. a.** Three new regions of density are present on the tau PHF following 3-hour incubation with EGCG, as compared to untreated PHFs. The most prominent density (Site 1, left) is in the polar cleft near the intersection of the two protofilaments of the fibril. The two minor density regions (Site 2, middle; Site 3, right) are also shown. **b.** EGCG modeled into each new density. Both Site 2 and Site 3 border lysine residues (K343 and K347), with the most electronegative oxygen (4' on the EGCG D ring) modeled interacting with the lysine residues. Though, these are speculative models for Sites 2 and 3, as the density is not sufficient for accurate modeling. Sites 2 and 3 are also less likely binding sites, given their buried surface area to the fibril are lower compared to Site 1: 227 Å^2^ (Site 1) versus 34 Å^2^ (Site 2) and 38 Å^2^ (Site 3). c. Density map of tau PHF without the addition of EGCG, highlighting the lack of density at Sites 1, 2, and 3.


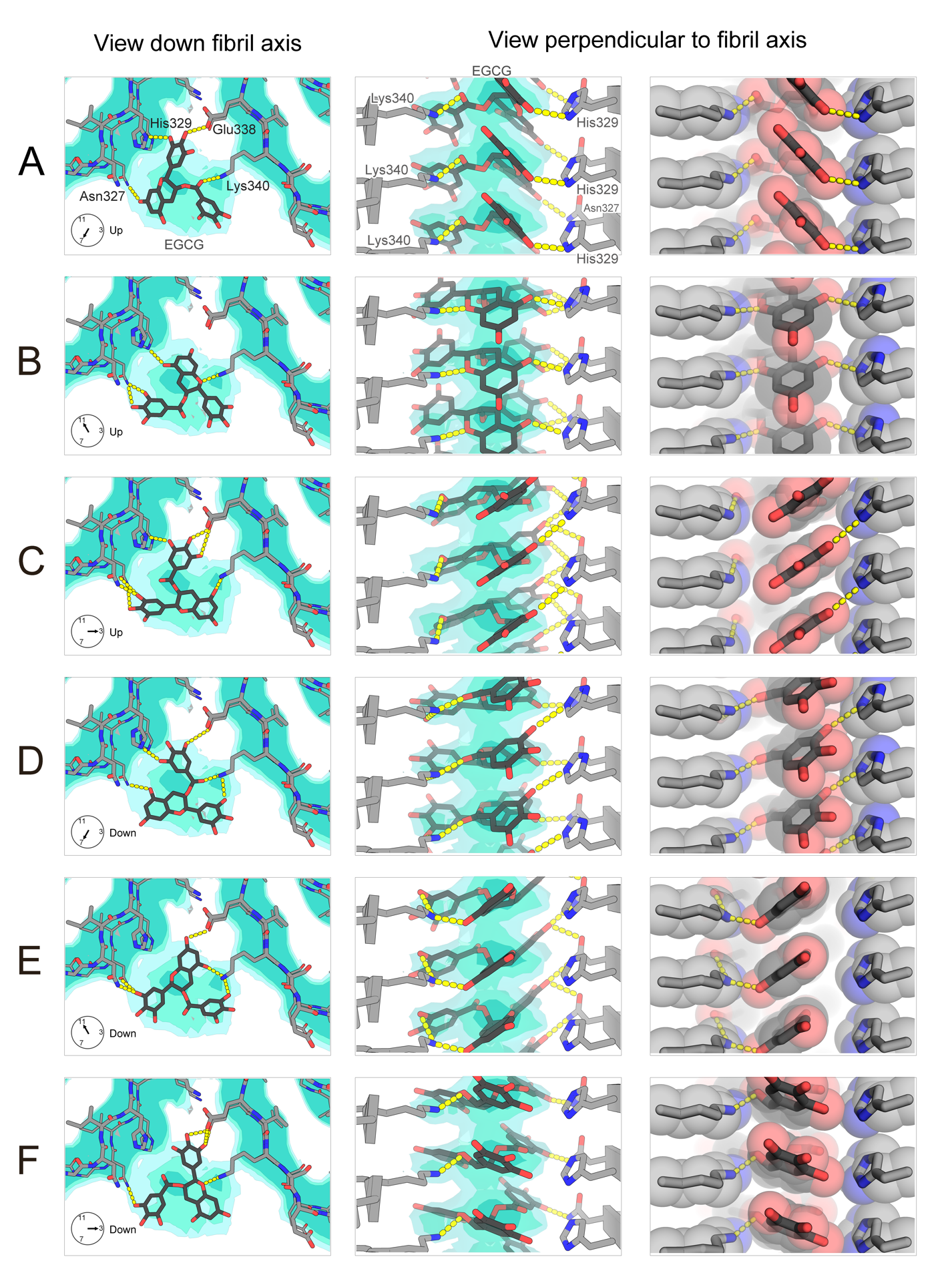


**Supplementary Fig. 6: Assessment of the most probable orientation of EGCG binding to tau PHFs.** Owing to limited resolution, it is possible to fit an EGCG molecule in multiple orientations in the Site 1 density of the 3-hour tau PHF EGCG map. We considered a comprehensive set of six orientations of the EGCG molecule in which the three aromatic branches of the EGCG molecule were alternatively fit in the three lobes of density (denoted 3, 7, and 11 o’clock). An additional three analogous orientations were modeled with the EGCG molecule flipped upside down (denoted up, down). Each of the six alternative conformations (A-F) is presented, with views down the fibril axis (left), perpendicular to the fibril axis (middle), and space filling model (right). Dashed yellow lines indicate hydrogen-bonding. Conformation C displays slightly superior due to hydrogen-bonding, π-π stacking and density fitting. Other conformations cannot be excluded from consideration. Model statistics for each conformation are detailed in Supplementary Table 2.


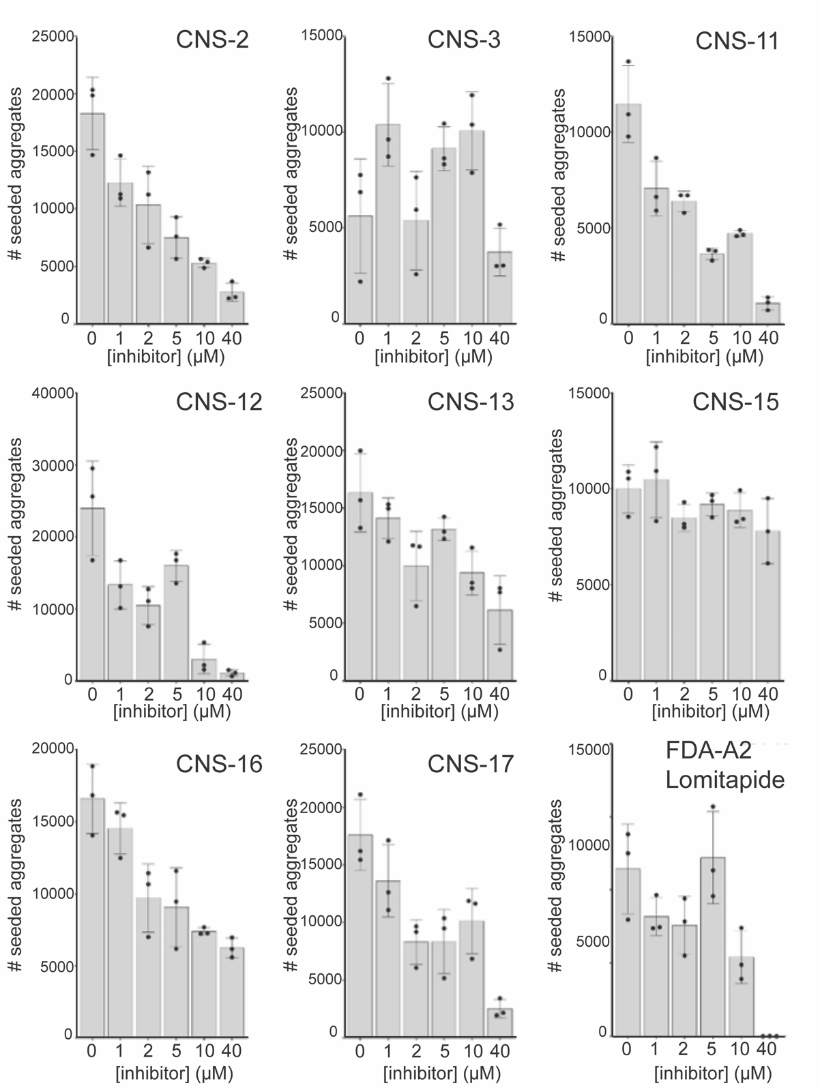


**Supplementary Fig. 7: Dose-dependent effects of lead compounds in tau biosensor cells.** Nine compounds identified from the *in silico* screen using the tau PHF EGCG pharmacophore showed a >50% reduction in tau seeding activity in biosensor cells at 10 µM inhibitor concentration (Fig. 3c). Brain derived tau PHFs were tested with various concentrations of each inhibitor compound, as shown above. Apart from CNS-3 and CNS-15, all nine compounds displayed dose-dependent efficacy in reducing intracellular tau seeding. Each condition was tested in triplicate, error bars represent ±SD of n=3 technical replicates.


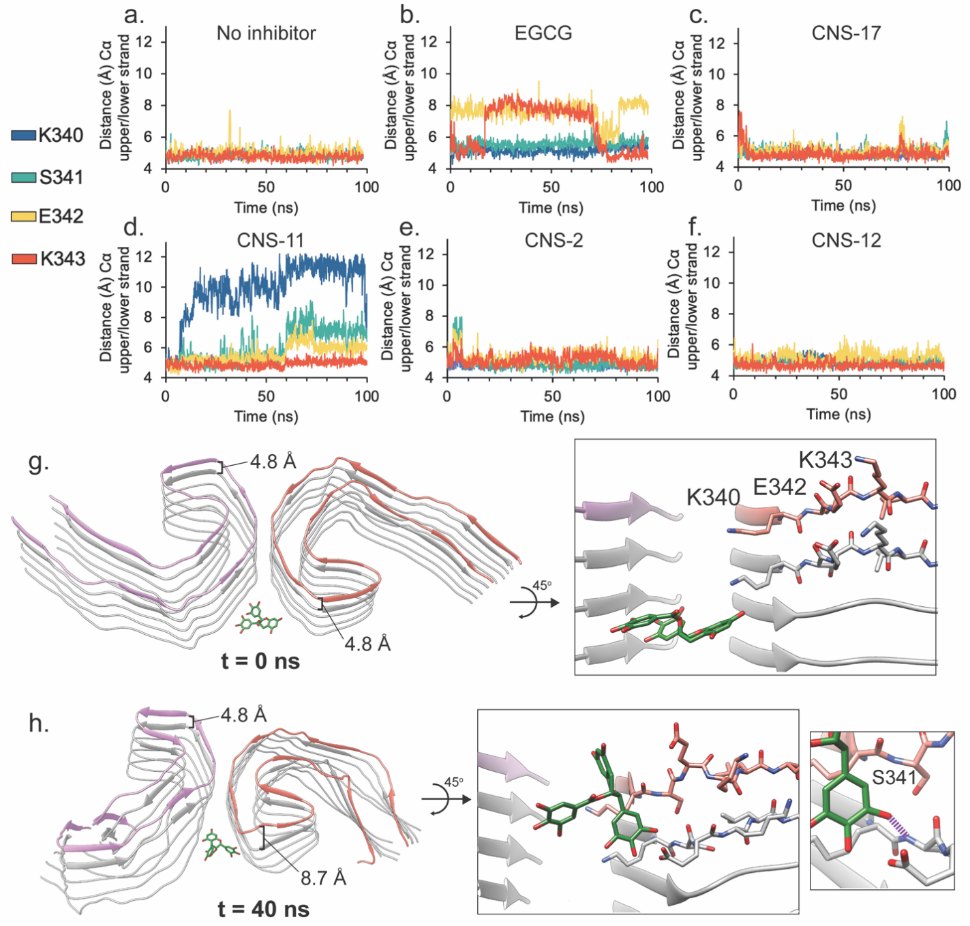


**Supplementary Fig. 8: Molecular dynamics simulations of inhibitor bound AD-tau fibrils.** Fibrils with inhibitors docked in the Site 1 EGCG binding pocket were subjected to 100 ns MD simulations and the inter-strand spacing of residues Lys340-Lys343 were plotted as a function of time. **a**. Without inhibitor bound, fibrils remain stable with intact layers characterized by the 4.8 Å spacings. **b**. A single docked EGCG molecule increases inter-strand spacing at Glu342 and Lys343 to ~8 Å. **c-e.** Effects of CNS-set inhibitors on inter-strand spacing. Only CNS-11 perturbs the inter-strand spacing. **g-h**. Docking models of EGCG (**g**) before and (**h**) after 40 ns MD simulations. Note the increase in inter-strand spacing centered at Lys340 and at the adjacent residues Ser341 and Glu342. **h.** Throughout the simulation, EGCG forms a hydrogen bond with the backbone amide of S341, likely contributing to the destabilization of adjacent resides.

**
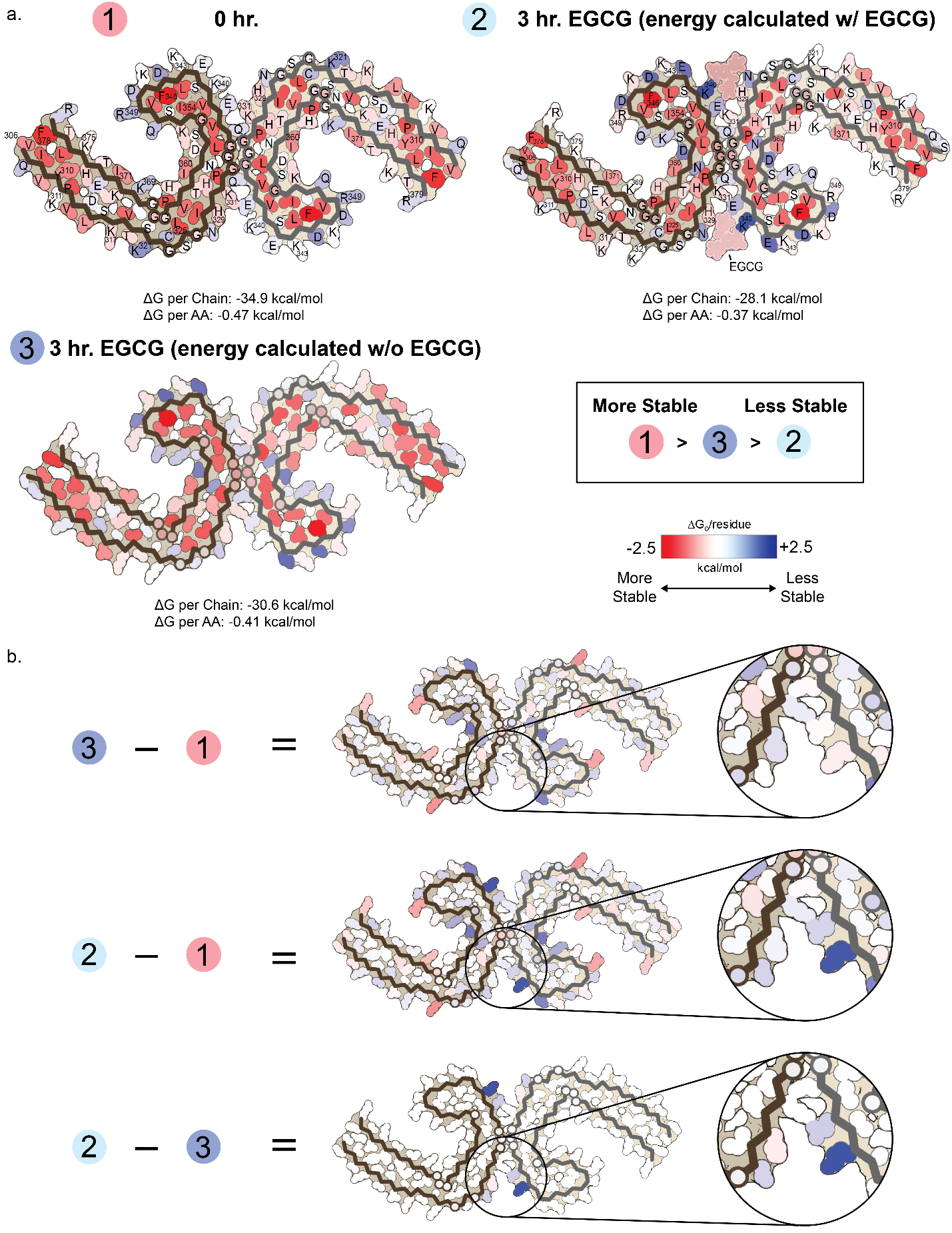
Supplementary Fig. 9: Solvation energy calculations of tau PHF and EGCG-PHF structures. a.** Solvation energy calculations of tau PHF structures at 0-hour EGCG incubation (“1”), 3-hour EGCG incubation calculated with EGCG molecules included in the model (“2”), and 3-hour EGCG incubation with EGCG not modeled (“3”). **b.** Difference energy maps. Subtraction of model #1 and model #3 (top) highlights little change in the solvation energy of the residues adjacent to the EGCG binding site on the PHF. However, subtraction of model #1 from #2 (middle) or model #3 from #2 (bottom) shows a large shift in solvation energy of Lys340 at the EGCG binding site. As model #2 is calculated with the EGCG molecule present, these results indicate that the presence of EGCG within the binding pocket is significantly destabilizing Lys340.


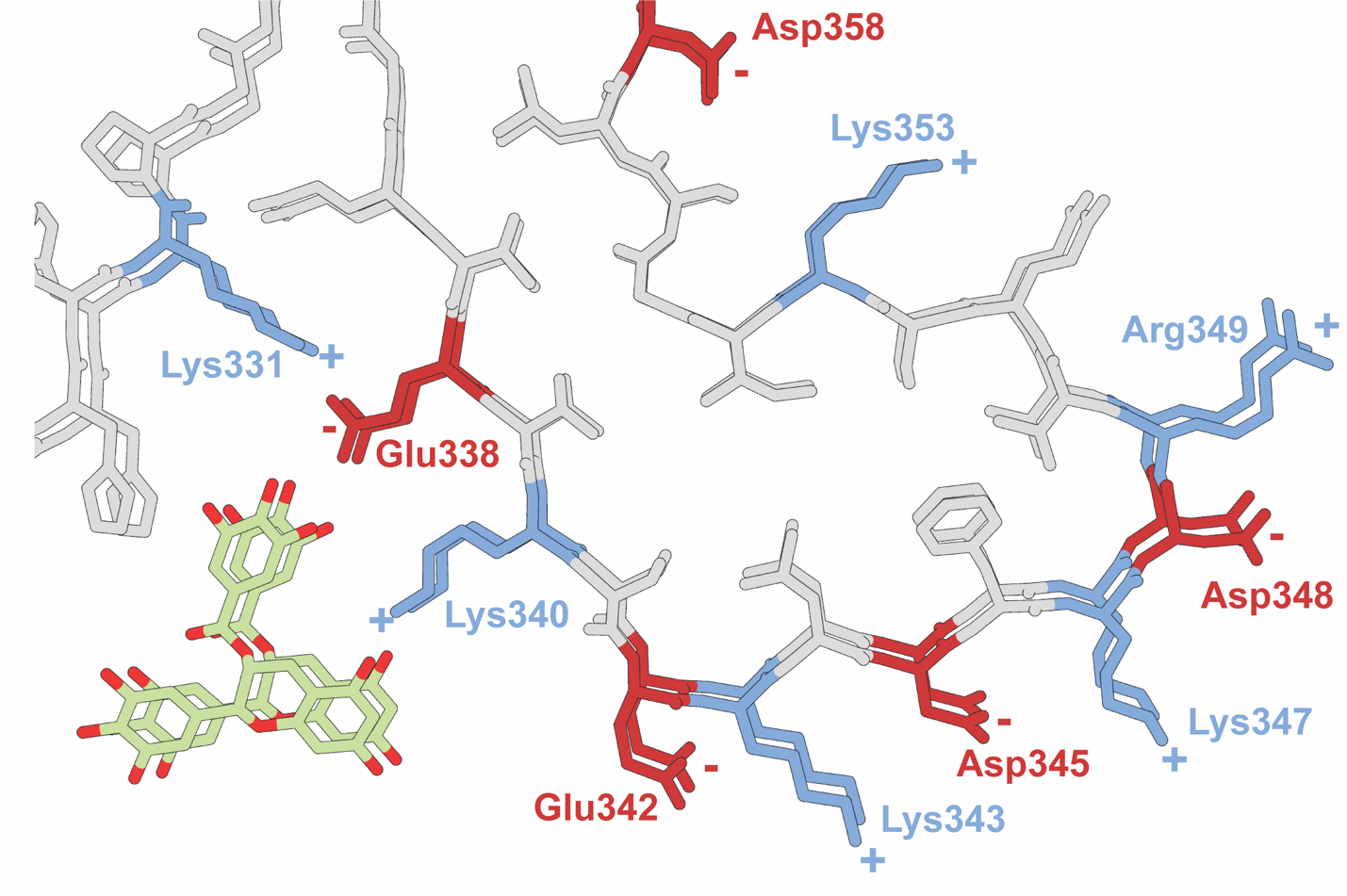


**Supplementary Fig. 10: Role of charge pairing in fibril stability.** Cross-sectional view of the 3-hour EGCG-tau-PHF complex structure showing alternating negatively (red) and positively (blue) charged sidechains of the β-helix segment. Stabilization of the fibril structure is provided by pairing of neighboring oppositely charged residues. By forming a H-bond with Lys340 and Glu338 of tau (Site 1), bound EGCG diminishes the effect of ion pair-mediated charge stabilization with neighboring charged residues, thereby increasing inter-layer repulsion of unpaired charges in neighboring layers. This repulsion is expected to weaken inter-layer bonding, favoring fibril disruption. Similar charge repulsion may also occur at EGCG Sites 2 and 3 (adjacent to Lys 343 and Lys 347, respectively), as EGCG binding may disrupt interaction with nearby Glu342/Asp/345/Aps348.


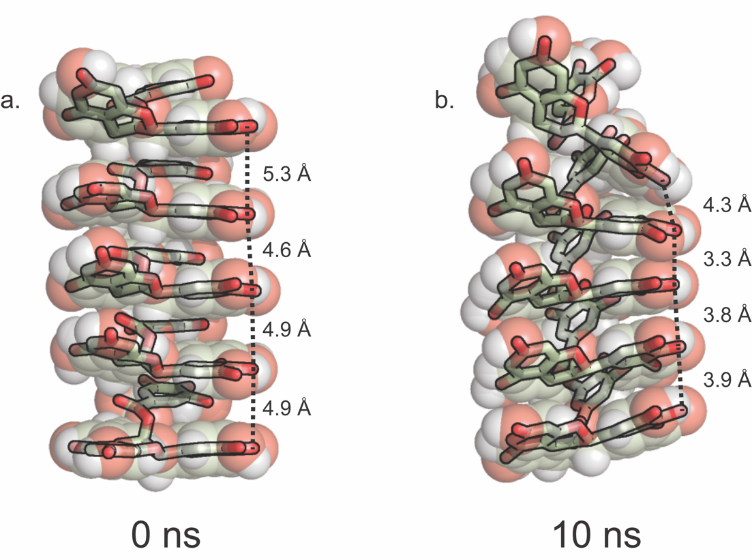


**Supplementary Fig. 11: Molecular dynamics simulation of stacked EGCG molecules. a.** A stack of EGCG molecules, as modeled in the 3-hour EGCG-tau PHF complex structure, consists of repeated layers of EGCG in a planar conformation spaced ~4.8 Å apart at the 0-nanosecond simulation timepoint. b. At 10 nanoseconds, a curvature is observed in the EGCG stack, as the molecules optimize van der Waals contacts between aromatic rings. This results in reduced interlayer spacing to ~3.6 Å for part of the stack, producing a curved conformation.


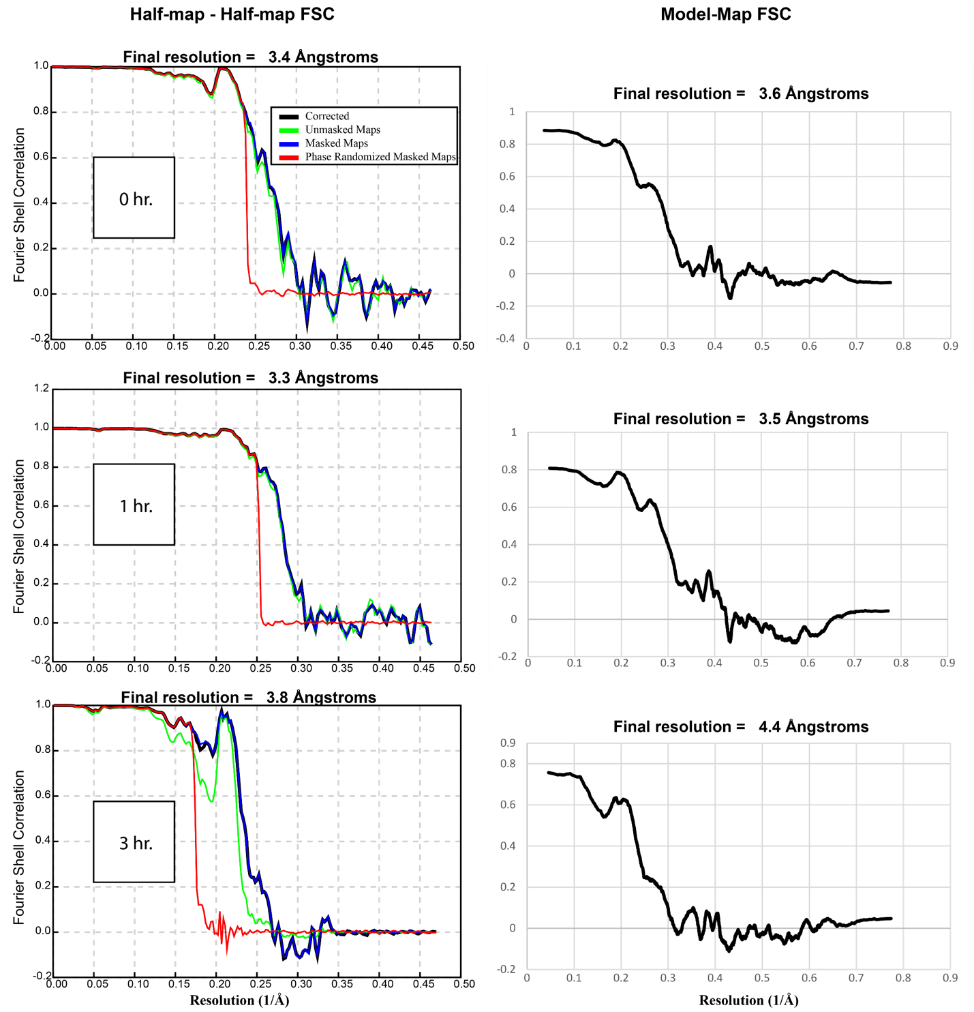


**Supplementary Fig. 12: Fourier shell correlation curves for cryoEM models and maps.**

|  | 0 hr. EGCG | 1 hr. EGCG | 3 hr. EGCG |
| --- | --- | --- | --- |
| **Data collection** | | | |
| Magnification | ×130,000 | ×130,000 | ×130,000 |
| Defocus range (um) | 1.8-2.25 | 1.8-2.25 | 1.8-2.25 |
| Voltage (kV) | 300 | 300 | 300 |
| Camera | K3 BioQuantum | K3 BioQuantum | K2 Summit  (Quantum LS) |
| Frame exposure time (s) | 0.05 | 0.05 | 0.2 |
| # movie frames | 48 | 48 | 30 |
| Total electron dose ( e-/Å^2^) | 80 | 58 | 19 |
| Pixel size (Å) | 1.078 | 1.078 | 1.064 |
| **Reconstruction** | | | |
| Box size (pixel) | 320 | 686 (2D/3D)  320 (3D) | 686 (2D/3D)  432 (3D) |
| Inter-box distance (Å) | 34.5 | 74.0 (686 pixel box)  34.5 (320 pixel box) | 74.0 (686 pixel box)  46.2 (432 pixel box) |
| # micrograph collected | 2,651 | 2,849 | 6,988 |
| # segments extracted | 116,322 | 103,251 (2D/3D – 686 pixel box)  166,486 (3D – 320 pixel box) | 111,775 (686 pixel box) |
| # segments after Class2D/Class3D | 89,325 | 89,380 (686 pixel box) | 57,540 (2D/3D – 686 pixel box)  137,000 (3D - 432 pixel box) |
| # final segments after Class3D | 33,752 | 20,759 | 29,145 |
| Resolution (Å) | 3.4 | 3.3 | 3.8 |
| Map sharpening B-factor (Å^2^) | -120.0 (adhoc) | -121.8 (manual) | -120.0 (adhoc) |
| Helical rise (Å) | 2.40 | 2.40 | 2.40 |
| Helical twist (°) | 179.47 | 179.47 | 179.46 |
| Point Group | C_1_ | C_1_ | C_1_ |
| **Atomic model** | | | |
| # non-hydrogen atoms | 5680 | 5680 | 6070 |
| # protein residues | 740 | 740 | 740 |
| R.m.s.d. bonds (Å) | 0.007 | 0.007 | 0.007 |
| R.m.s.d. angles (°) | 1.042 | 1.042 | 1.146 |
| Molprobity clashscore, all atoms | 13.9 | 13.9 | 13.9 |
| Molprobity score | 2.15 | 2.15 | 2.14 |
| Poor rotamers (%) | 0 | 0 | 0 |
| Ramachandran outliers (%) | 0 | 0 | 0 |
| Ramachandran allowed (%) | 8.33 | 8.33 | 8.22 |
| Ramachandran favored (%) | 91.67 | 91.67 | 91.78 |
| Cβ deviations > 0.25 Å (%) | 0 | 0 | 0 |
| Bad bonds (%) | 0 | 0 | 0 |
| Bad angles (%) | 0 | 0 | 0 |
| PDB ID: | 7UPE | 7UPF | 7UPG |
| EMDB ID: | EMD-26663 | EMD-26664 | EMD-26665 |

**Supplementary Table 1: CryoEM data collection, model refinement, and validation statistics.**

|  | Pose A | Pose B | Pose C | Pose D | Pose E | Pose F | Notes |
| --- | --- | --- | --- | --- | --- | --- | --- |
| CC_mask | 0.662 | 0.663 | 0.664 | 0.664 | 0.664 | 0.664 | Tau+EGCG atoms fit the map well in all 6 models. All fits are similar quality. 6070 atoms/model. |
| CC_EGCG_ center molecule in stack | 0.51 | 0.62 | 0.54 | 0.61 | 0.56 | 0.53 | Small differences in EGCG fit. |
| Clashscore Tau+EGCG all atoms | 13.2 | 16.0 | 13.9 | 14.0 | 13.2 | 13.9 | No serious clashes in any of the 6 models. |
| Clashscore pharmacophore | 1.5 | 26.5 | 7.4 | 8.8 | 1.5 | 7.4 | Clashscores for pharmacophore are OK. |
| EGCG Solvent Accessible Surface Area buried (Å^2^) | 496 | 468 | 491 | 473 | 488 | 486 | EGCG is about 75% buried in all cases. Differences are relatively small. |
| EGCG Sc | 0.75 | 0.67 | 0.77 | 0.70 | 0.68 | 0.63 | Shape complementarity is high in all cases. |
| EGCG Hydrogen bond count | 4 | 4 | 10 | 5 | 5 | 5 | Pose C exhibits the largest H-bonding network. |
| EGCG Unsatisfied H-bond donors/ acceptors (central chain) | 7 | 7 | 4 | 6 | 7 | 6 | Pose C exhibits the fewest unsatisfied H-bond donors/acceptor atoms. |
| ΔG°_sol_ EGCG central (kcal/mol) | -0.98 | -1.31 | -0.20 | -0.60 | -1.22 | -1.02 | Pose C exhibits least stableΔG°_sol_ because it buries the most polar atoms. |
| ΔG°_sol_ EGCG tip 1 (kcal/mol) | -0.79 | -1.00 | -0.03 | +0.57 | -0.52 | -0.13 |  |
| ΔG°_sol_ EGCG tip 2 (kcal/mol) | -0.23 | -0.37 | +0.55 | -0.32 | -0.83 | -0.72 |  |
| ΔG°_sol_ Tau central chain (kcal/mol) | -28.7 | -29.2 | -27.9 | -28.7 | -29.0 | -28.6 | Pose C destabilizes Tau most strongly because it buries the most polar atoms. |
| ΔG°_sol_ Tau tip1 chain (kcal/mol) | -20.9 | -18.3 | -17.8 | -17.2 | -18.3 | -17.8 |  |
| ΔG°_sol_ Tau tip2 chain (kcal/mol) | -16.5 | -17.5 | -15.7 | -17.2 | -17.1 | -16.9 |  |

**Supplementary Table 2: Model statistics for EGCG binding conformations.**

| **Compound** | **Chemical name** | **ChemBridge ID #** |
| --- | --- | --- |
| CNS-1 | 10,12-dimethyl-10,12-dihydro-7H,11H-benzo[de]imidazo[4',5':5,6]benzimidazo[2,1-a]isoquinoline-7,11-dione | 7850915 |
| CNS-2 | 2-(7-phenyl[1,2,4]triazolo[1,5-a]pyrimidin-2-yl)-1H-isoindole-1,3(2H)-dione | 7220009 |
| CNS-3 | 6-chloro-3-[5-(3-fluorophenyl)-1,3,4-oxadiazol-2-yl]-2H-chromen-2-one | 6624890 |
| CNS-4 | 4-(4-fluorophenyl)-2-methyl-5-oxo-N-2-pyridinyl-1,4,5,6,7,8-hexahydro-3-quinolinecarboxamide | 6317572 |
| CNS-5 | 4-(3-methoxyphenyl)-2-methyl-5-oxo-N-2-pyridinyl-1,4,5,6,7,8-hexahydro-3-quinolinecarboxamide | 6327117 |
| CNS-6 | 2-methyl-4-(2-methylphenyl)-N-(6-methyl-2-pyridinyl)-5-oxo-1,4,5,6,7,8-hexahydro-3-quinolinecarboxamide | 6343767 |
| CNS-7 | 9-(1,3-benzodioxol-5-yl)-3,3,6,6-tetramethyl-3,4,6,7,9,10-hexahydro-1,8(2H,5H)-acridinedione | 6218692 |
| CNS-8 | 2-methyl-N-(6-methyl-2-pyridinyl)-5-oxo-4-(3-pyridinyl)-1,4,5,6,7,8-hexahydro-3-quinolinecarboxamide | 6347615 |
| CNS-9 | N-(tert-butyl)-N-(difluoromethyl)-4-methylbenzenesulfonamide | 6933498 |
| CNS-10 | 6-bromo-3-(3,4-dihydro-2(1H)-isoquinolinylcarbonyl)-2H-chromen-2-one | 6991358 |
| CNS-11 | N-mesityl-2-(3-oxoindeno[1,2,3-de]phthalazin-2(3H)-yl)acetamide | 7119834 |
| CNS-12 | 1-(3-chlorophenyl)-3-(3,5-dimethylphenyl)tetrahydro-1H-thieno[3,4-d]imidazol-2(3H)-one 5,5-dioxide | 7953084 |
| CNS-13 | N-(4-methyl-2-pyridinyl)-2-[(5-methyl-5H-[1,2,4]triazino[5,6-b]indol-3-yl)thio]acetamide | 5917242 |
| CNS-14 | 2-methyl-4-(4-methylphenyl)-N-(4-methyl-2-pyridinyl)-5-oxo-1,4,5,6,7,8-hexahydro-3-quinolinecarboxamide | 6314166 |
| CNS-15 | 2-methyl-5-oxo-N-2-pyridinyl-4-(2-thienyl)-1,4,5,6,7,8-hexahydro-3-quinolinecarboxamide | 6314475 |
| CNS-16 | N-(6-methyl-2-pyridinyl)-2-oxo-2H-chromene-3-carboxamide | 5535268 |
| CNS-17 | N-[3-(trifluoromethyl)phenyl]-10H-phenothiazine-10-carboxamide | 6870810 |
| CNS-18 | 2-phenyl-4,5,6,7-tetrahydro-8H-cyclopenta[d]pyrazolo[1,5-a]pyrimidin-8-one | 7071662 |
| CNS-19 | N-[5-(3-methyl-4-oxo-3,4-dihydro-1-phthalazinyl)-2-(1-piperidinyl)phenyl]acetamide | 7520752 |
| CNS-20 | 5,14-dioxo-5,14-dihydrobenzo[5,6]indolo[1,2-b]isoquinoline-13-carbonitrile | 7663820 |
| CNS-21 | N-(2-methoxy-5-methylphenyl)-2-(2-oxobenzo[cd]indol-1(2H)-yl)acetamide | 7678251 |
| CNS-22 | 3-(1H-benzimidazol-1-yl)-1-(2,4-dimethylphenyl)-2,5-pyrrolidinedione | 7729218 |
| CNS-23 | 3-(2,3-dihydro-1,4-benzodioxin-2-yl)-6-phenyl-7H-[1,2,4]triazolo[3,4-b][1,3,4]thiadiazine | 7958423 |
| CNS-24 | 2-[3-(3-methylphenyl)-1,2,4-oxadiazol-5-yl]-N-(3-pyridinylmethyl)benzamide | 9002758 |
| CNS-25 | N-(2-methoxyphenyl)-3-oxo-3H-benzo[f]chromene-2-carboxamide | 5876327 |
| FDA-R-1 | Docarpamine |  |
| FDA-R-2 | Gliquidone |  |
| FDA-R-3 | Arteflene |  |
| FDA-R-4 | Naratriptan |  |
| FDA-R-5 | Fudosteine |  |
| FDA-R-6 | Lanoconazole |  |
| FDA-R-7 | Nizatidine |  |
| FDA-R-8 | Acetohexamide |  |
| FDA-R-9 | Phytonadione |  |
| FDA-R-10 | Sarecycline |  |
| FDA-R-11 | Tubocurarine |  |
| FDA-R-12 | Phentermine |  |
| FDA-R-13 | Levobupivacaine |  |
| FDA-R-14 | Isoetharine |  |
| FDA-R-15 | Prolixin decanoate |  |
| FDA-R-16 | Sulfamethazine |  |
| FDA-R-17 | Isosorbide Mononitrate |  |
| FDA-R-18 | Travoprost |  |
| FDA-R-19 | Natamycin |  |
| FDA-R-20 | Phenylbutazone |  |
| FDA-R-21 | Agrimophol |  |
| FDA-R-22 | Trimetrexate |  |
| FDA-R-23 | Sodium chromoglycate |  |
| FDA-R-24 | Imipramine |  |
| FDA-R-25 | Zolmitriptan |  |
| FDA-A-1 | Ergotamine |  |
| FDA-A-2 | Lomitipide |  |
| FDA-A-3 | Dihydroergotamine nasal |  |
| FDA-A-4 | Temoporfin |  |
| FDA-A-5 | Tasosartan |  |
| FDA-A-6 | Candesartan |  |
| FDA-A-7 | Everolimus |  |
| FDA-A-8 | Dihydroergocristine |  |
| FDA-A-9 | Dutasteride |  |
| FDA-A-10 | Amlexanox |  |
| FDA-A-11 | Teniposide |  |
| FDA-A-12 | Saprisartan |  |
| FDA-A-13 | Ingenol mebutate |  |
| FDA-A-14 | MK-8228 |  |
| FDA-A-15 | Eltrombopag |  |
| FDA-A-16 | Conivaptan |  |
| FDA-A-17 | Irbesartan |  |
| FDA-A-18 | Fenquizone |  |
| FDA-A-19 | Cyclothiazide |  |
| FDA-A-20 | Suvorexant |  |
| FDA-A-21 | Raltegravir |  |
| FDA-A-22 | Ajmalicine |  |
| FDA-A-23 | Dihydroergotamine |  |
| FDA-A-24 | Idarubicin |  |
| FDA-A-25 | Daunorubicin |  |

**Supplementary Table 3:** List of compounds identified from the Chembridge CNS-Set and FDA approved compound libraries by *in silico* screening using the tau PHF EGCG pharmacophore.

| **Compound** | **Chemical name** | **H-bond donors** | **H-bond acceptors** | **Molecular weight** | **XLogP3** | **Rotatable bonds** | **Polar surface area (Å^2^)** |
| --- | --- | --- | --- | --- | --- | --- | --- |
| **EGCG** | Epigallocatechin gallate | 8 | 11 | 458.4 | 1.2 | 4 | 197 |
| **CNS-11** | N-mesityl-2-(3-oxoindeno[1,2,3-de]phthalazin-2(3H)-yl)acetamide | 1 | 3 | 395 | 4.6 | 3 | 62 |
| **CNS-12** | 1-(3-chlorophenyl)-3-(3,5-dimethylphenyl)tetrahydro-1H-thieno[3,4-d]imidazol-2(3H)-one 5,5-dioxide | 0 | 3 | 390.0 | 3.3 | 2 | 66.1 |
| **CNS-17** | N-[3-(trifluoromethyl)phenyl]-10H-phenothiazine-10-carboxamide | 1 | 5 | 386.4 | 5.3 | 1 | 57.6 |
| **CNS-2** | 2-(7-phenyl[1,2,4]triazolo[1,5-a]pyrimidin-2-yl)-1H-isoindole-1,3(2H)-dione | 0 | 5 | 341.3 | 2.6 | 2 | 80.5 |

**Supplementary Table 4: Table of biophysical properties for EGCG and lead compounds.** Compounds judged for drug-likeness based on Lipinski’s and Verber’s rules (H-bond donors < 5, H-bond acceptors < 10, MW < 500 kDa, octanol-water partition coefficient (LogP) < 5, rotatable bonds < 10, polar surface area < 140 Å^2^. Green indicates rule satisfaction, red indicates rule violation. EGCG is in violation of several of these criteria.
