# Supplementary material for: Structure-based discovery of small molecules that disaggregate Alzheimer’s disease tissue derived tau fibrils in vitro
Source: Nat Commun. 2022 Sep 16;13:5451. doi: 10.1038/s41467-022-32951-4 (PMC9481533; doi:10.1038/s41467-022-32951-4)
Supplement: Supplementary file 3 — Reporting Summary [file 41467_2022_32951_MOESM3_ESM.pdf]

nature portfolio

Corresponding author(s): David S. Eisenberg

Last updated by author(s): Aug 3, 2022

Reporting Summary

Nature Portfolio wishes to improve the reproducibility of the work that we publish. This form provides structure for consistency and transparency in reporting. For further information on Nature Portfolio policies, see our Editorial Policies and the Editorial Policy Checklist.

Statistics

For all statistical analyses, confirm that the following items are present in the figure legend, table legend, main text, or Methods section.

n/a	Confirmed	
☐	☒	The exact sample size (n) for each experimental group/condition, given as a discrete number and unit of measurement
☐	☒	A statement on whether measurements were taken from distinct samples or whether the same sample was measured repeatedly
☐	☒	The statistical test(s) used AND whether they are one- or two-sided<br>*Only common tests should be described solely by name; describe more complex techniques in the Methods section.*
☒	☐	A description of all covariates tested
☒	☐	A description of any assumptions or corrections, such as tests of normality and adjustment for multiple comparisons
☒	☐	A full description of the statistical parameters including central tendency (e.g. means) or other basic estimates (e.g. regression coefficient) AND variation (e.g. standard deviation) or associated estimates of uncertainty (e.g. confidence intervals)
☐	☒	For null hypothesis testing, the test statistic (e.g. F, t, r) with confidence intervals, effect sizes, degrees of freedom and P value noted<br>*Give P values as exact values whenever suitable.*
☒	☐	For Bayesian analysis, information on the choice of priors and Markov chain Monte Carlo settings
☒	☐	For hierarchical and complex designs, identification of the appropriate level for tests and full reporting of outcomes
☒	☐	Estimates of effect sizes (e.g. Cohen's d, Pearson's r), indicating how they were calculated

Our web collection on statistics for biologists contains articles on many of the points above.

Software and code

Policy information about availability of computer code

Data collection	Single particle data collection was managed using the Leginon 3.3 software.
Data analysis	Cryo-EM data were processed using a combination of Unblur 1.00, CTFFIND 4.1.8, mag_distortion_estimate 0.0.0, EMAN 2.2 e2helixboxer.py, RELION 3.1. Phenix 1.2 (auto_sharpen, real_space_refine), COOT 0.9.8.2, ImageJ 2.3.0 was used for biosensor cell counting using a custom script available upon request; data was processed using Microsoft Excel 2020 v. 16.6. AutoDock Vina 1.1.2 and RosettaLigand (Rosetta version 3.10) were used for in silico docking. MD simulations were performed using GROMACS 2018.

For manuscripts utilizing custom algorithms or software that are central to the research but not yet described in published literature, software must be made available to editors and reviewers. We strongly encourage code deposition in a community repository (e.g. GitHub). See the Nature Portfolio guidelines for submitting code & software for further information.

Data

Policy information about availability of data

All manuscripts must include a data availability statement. This statement should provide the following information, where applicable:

- Accession codes, unique identifiers, or web links for publicly available datasets
- A description of any restrictions on data availability
- For clinical datasets or third party data, please ensure that the statement adheres to our policy

Structure coordinates and map files are deposited into the Worldwide Protein Data Bank (wwPDB) and the Electron Microscopy Data Bank (EMDB) with the following accession codes: PDB ID 7UPE/EMD-26663 (no EGCG), PDB ID 7UPF/EMD-26664 (1-hour), and PDB ID 7UPG/EMD-26665 (3-hour).

Human research participants

Policy information about studies involving human research participants and Sex and Gender in Research.

Reporting on sex and gender	Human autopsy samples were obtained by the Mayo Clinic Brain Bank and UCLA Pathology Department according to DHHS regulations from patients consenting to autopsy. Samples were provided to the researchers of this study as anonymized tissues.
Population characteristics	Anonymous
Recruitment	Anonymous
Ethics oversight	IRB approval not required for anonymous samples. DHHS regulations from patients consenting to autopsy were followed.

Note that full information on the approval of the study protocol must also be provided in the manuscript.

Field-specific reporting

Please select the one below that is the best fit for your research. If you are not sure, read the appropriate sections before making your selection.

☒ Life sciences ☐ Behavioural & social sciences ☐ Ecological, evolutionary & environmental sciences

For a reference copy of the document with all sections, see nature.com/documents/nr-reporting-summary-flat.pdf

Life sciences study design

All studies must disclose on these points even when the disclosure is negative.

Sample size	A sample size of 3 experimental replicates was selected for the analysis of inhibitor efficacy based on the minimum sample size that is required to detect a 50% decrease in seeding with sufficient power. For structure determination, brain extracts from the frontal cortex of 5 brain tissue donors with AD were procured and used for fibril purification. Tissue from only 1 of the 5 donors yielded fibrils in sufficient quantity and purity from cryoEM structure determination.
Data exclusions	All data was included in all of our analyses.
Replication	All experiments were performed as experimental replicates of 3 unless otherwise stated in the text.
Randomization	For image analysis, samples were randomly assigned a number code that was used to identify the sample treatment, and data were decoded after data collection and image analysis was completed.
Blinding	Image analysis was performed blindly using numerical coding and unbiased script-based analysis. The same researcher who prepared the samples and performed the experiments analyzed the data.

Reporting for specific materials, systems and methods

We require information from authors about some types of materials, experimental systems and methods used in many studies. Here, indicate whether each material, system or method listed is relevant to your study. If you are not sure if a list item applies to your research, read the appropriate section before selecting a response.

Materials & experimental systems

n/a	Involved in the study	
☐	☒	Antibodies
☐	☒	Eukaryotic cell lines
☒	☐	Palaeontology and archaeology
☒	☐	Animals and other organisms
☒	☐	Clinical data
☒	☐	Dual use research of concern

Methods

n/a	Involved in the study	
☒	☐	ChIP-seq
☒	☐	Flow cytometry
☒	☐	MRI-based neuroimaging

Antibodies

Antibodies used	GT38 antibody for pathologic Alzheimer's disease tau was a gift from the lab of Virginia Less and University of Pennsylvania. AT8 antibody (anti-phospho-tau) was purchased from ThermoFisher (cat # MN1020; lot # UL2906281Z).
Validation	GT38 was validated via dot blot, ELISA, and Western blot to bind to AD tau fibrils, but not other pathological or recombinant tau fibrils (or other protein fibrils). AT8 was not validated beyond the manufacturer's validation (per ThermoFischer: AT8 was verified by cell treatment to ensure that the antibody binds to the antigen stated.).

Eukaryotic cell lines

Policy information about cell lines and Sex and Gender in Research

Cell line source(s)	HEK293T tau biosensor cells were obtained from Marc Diamond's lab at UTSW.
Authentication	HEK293T tau biosensor cells were authenticated by STR analysis.
Mycoplasma contamination	HEK293T tau biosensor cells tested negative for mycoplasma contamination.
Commonly misidentified lines (See ICLAC register)	None.
